# Puncture Site Bleeding Complications of Peripheral Nerve Block in Patients Taking Antithrombotic and Anticoagulant Drugs: An Umbrella Review

**DOI:** 10.31661/gmj.v12i.2958

**Published:** 2023-10-30

**Authors:** Ahmad Rastgarian, Khatereh Dehghani, Shahram Shafa, Mohammad Sadegh Sanie Jahromi, Mansour Deylami, Soha Azizi, Mohammad Zarenezhad, Kaveh Hedayati Emami, Vahid Rahmanian, Tayyebeh Zarei, Navid Kalani

**Affiliations:** ^1^ Critical Care and Pain Management Research Center, Jahrom University of Medical Sciences, Jahrom, Iran; ^2^ Department of Cardiology, Jahrom University of Medical Sciences, Jahrom, Iran; ^3^ Department of Orthopedics, Jahrom University of Medical Sciences, Jahrom, Iran; ^4^ Department of Anesthesiology and Critical Care, Faculty of Medicine, Golestan University of Medical Sciences, Gorgan, Iran; ^5^ Student Research Committee, Jahrom University of Medical Sciences, Jahrom, Iran; ^6^ Legal Medicine Research Center, Legal Medicine Organization, Tehran, Iran; ^7^ Department of Anesthesiology and Critical Care, Imam Khomeini Hospital, Tehran University of Medical Sciences, Tehran, Iran; ^8^ Department of Public Health, Torbat Jam Faculty of Medical Sciences, Torbat Jam, Iran; ^9^ Department of Anesthesiology, Anesthesiology, Critical Care and Pain Management Research Center, Hormozgan University of Medical Sciences, Bandar Abbas, Iran; ^10^ Research Center for Social Determinants of Health, Jahrom University of Medical Sciences, Jahrom, Iran

**Keywords:** Peripheral Nerve Block, Puncture Site Bleeding, Complications, Antithrombotic Agents

## Abstract

Background: While there are multiple guidelines for the management of bleeding complications and hematoma if being treated with antithrombotic and anticoagulant drugs, these risks are not yet stratified for procedures with regional anesthesia.Materials and Methods: This study was an umbrella review of systematic studies and meta-analysis based on PRISMA guidelines in databases of Scopus, PubMed, Medline, Cochrane Library, and Web of Science databases. Due to heterogeneity in evaluated outcomes and methods of studies, only the qualitative evidence synthesis was performed. AMSTAR checklist was used to assess the risk of bias in included systematic reviews.Results: After an extensive search of relevant studies, 971 primary cases were identified. Following a thorough screening process, 5 systematic reviews were selected. The evidence suggests that head and neck punctures generally do not result in bleeding complications, except for rare cases of hematoma associated with Infraclavicular brachial plexus block. A deep cervical plexus block is not recommended. Interscalene blocks have varying findings, with some studies reporting hematoma incidence and spinal injury, while others consider them low risk. Supraclavicular brachial plexus block might be associated with hemothorax and infraclavicular blocks are not favored by reviews. Axillary brachial plexus blocks have a minor incidence of hematoma. Abdomen blocks, TAP blocks, ilioinguinal blocks, and rectus sheath blocks carry a higher risk of hematoma. Pectoral nerve (PECS) blocks have a relatively high risk, while paravertebral and intercostal blocks are considered high risk, but further research is needed regarding paravertebral blocks.Conclusion: The available evidence from systematic reviews and studies suggests varying levels of risk for different blocks and procedures that should be considered before decision-making.

## Introduction

Complications of bleeding after regional anesthesia are rare, but when occurring, can have significant adverse outcomes [1][2]. Hematoma is a collection of blood in a part of the body but outside the blood vessels. These side effects are important in anesthesia interventions and anesthesia close to different nerves and most importantly the spinal cord [3][4]. Definitive determination of risk factors for the formation of hematoma and bloody puncture is more often occurring in patients undergoing nerve blocks with underlying coagulation problems, either due to disease or due to medical intervention to thin the blood [5][6]. Understanding the mechanisms of blood coagulation, medicinal properties of anticoagulant and antiplatelet drugs, as well as clinical studies involving patients under nerve block while receiving these drugs is very important in reducing the risk of bleeding and hematoma in patients [7][8]. Over the past twenty years, there have been advancements in medications to prevent blood clot formation in surgical patients. This raises a concern about how to safely perform regional anesthesia procedures while patients are taking these clot-preventing drugs to avoid severe bleeding problems [9][10][11].

For less potent anticoagulants, there is insufficient evidence, and the management of these conditions depends on the opinion of the anesthetist [12][13]. Therefore, it is controversial to perform nerve blocks in patients who receive antithrombotic and anticoagulant drugs. The previous systematic reviews are in the form of systematic reviews and panels of experts or clinical guidelines that have addressed various aspects of bleeding complications associated with peripheral nerve blocks in patients taking antithrombotic and anticoagulant drugs, while most of these studies share information for neuraxial blocks. By conducting an umbrella review of systematic studies and meta-analyses, our study seeks to synthesize and analyze the existing evidence to provide insights into the safety profiles of different nerve blocks in the context of anticoagulant use. For this reason, in this research, due to the importance of this issue and the expansion of the use of local and regional anesthesia, a review of systematic review studies about the risks ahead in patients using antithrombotic and antithrombotic drugs was conducted.

## Materials and Methods

This study was conducted as an umbrella review of systematic review studies and meta-analysis (in January 2023), based on the PRISMA guidelines [14]. The Patient/Intervention/Comparison/Outcome tool was used to structure the study. Based on this: The population is patients using anticoagulants and antiplatelets before peripheral nerve block. Intervention/exposure is the regional block and local anesthetic injection. The comparison was based on the rate of complications in each of the antithrombotic and antithrombotic drugs. The outcome was the rate of hemorrhagic complications (the number of complications in the total number of the studied population). All tasks listed below were performed by 2 independent researchers and disagreements were judged by another researcher. To obtain scientific evidence related to the complications of local anesthesia in patients using anticoagulant drugs from articles published in Web of Science, Cochrane Library, PubMed, Scopus, Embase, and Google Scholar search engine. The following sample PubMed pattern search strategy was used:

(complications OR adverse effects OR side effects OR risks OR bloody puncture OR hematoma OR bleeding) AND (local anesthesia Epidural anesthesia OR CSE anesthesia OR Peripheral nerve block OR brachial plexus block OR femoral nerve block OR intercostal nerve block OR sciatic nerve block OR axillary block OR Paravertebral block OR Retrobulbar block) AND (anticoagulant drugs OR anticoagulation OR blood thinners OR oral anticoagulants) with filters of Systematic Review. to obtain scientific evidence and documentation, references and sources of all included articles were examined for related records.

Eligibility Criteria

The criteria for the inclusion of studies in this research were English articles that compared the side effects of local anesthesia in patients taking anticoagulant drugs in the form of a to the full text of the article in English was one of the requirements to enter the study. Studies with any PRISMA-based systematic reviews were included in the study. Exclusion criteria include studies published in different languages except for English, those published after January 2023, not having full text, review studies and books, qualitative studies, and studies without mentioning the type of drug. Studies on patients whose type of anticoagulant or antiplatelet drug was not known were excluded. We excluded all studies on neuraxial anesthesia (spinal or epidural). Inadvertent vascular punctures were not counted as bleeding complications. Patients whose underlying disease is not known due to the use of anticoagulants were not included in the study. The latest version of the same guideline was confirmed to prevent duplicated studies inclusion.

Every article that was included in the final list underwent evaluation using a predefined checklist. The checklist includes primary scales such as the number of included studies, the type of drug used by patients, the reason for drug use, the reason for local anesthesia, local anesthetic drug and its dose, type of complication, and background information of the article including the name of the authors, country, year of publication, study place, study design.

Quality of Studies

We assessed the quality of all studies based on the Assessment of Multiple Systematic Review 2 (AMSTAR 2) checklist. Its online tool was used to evaluate the quality [15].

## Results

From the search of the studied sources, 971 primary cases were identified, after removing 58 duplicate cases, 931 articles were examined in terms of titles, and 254 unrelated cases were removed. Among the next 677 articles that were reviewed based on the article abstract, 598 irrelevant items were removed. Finally, 79 studies were reviewed in full text. Among them, 34 studies investigated the prophylactic role of starting antithrombotic treatment in patients who were to undergo a specific procedure, 31 cases were about sole neuraxial anesthesia and 9 were not systematic reviews. After removing these cases, finally, 5 systematic reviews were selected [16][17][18][19][20] (Table-1). The process of selecting studies is shown in Figure-1.

Results of Individual Studies

According to the Tsui et al. study, it’s important to avoid interpreting the lack of evidence in anticoagulated patients as a sign of reduced bleeding risk. This caution is relevant because current guidelines usually discourage administering these blocks to patients on anticoagulation therapy. While they have not cited the sources of synthesized shreds of evidence in their study, it seems that the used evidence is limited to case reports or case series studies. In Joubert et al.’s examination of twenty-four articles, they found that six were observational studies and eighteen were case descriptions. These studies focused on patients who had received different types of medications: antiplatelet drugs only (in 4 studies), anticoagulants alone (in 14 studies), or both types of medications (in 6 studies). Among the observational studies, there were a total of 80 instances of bleeding problems, like hematomas or minor bleeding at the injection site, out of 9,738 peripheral nerve blocks. In the case reports, 15 bleeding issues were documented in 50 peripheral nerve blocks. Most of these bleeding complications were associated with lumbar plexus blocks. In some cases, these complications were severe, requiring blood transfusions, catheter embolization, surgical exploration, and, sadly, one case resulted in a fatality. The overall estimated occurrence of bleeding complications was approximately 0.82%, with the range being from 0.64% to 1.0%. Kaye et al. study has provided valuable pieces of evidence in case of continuing or discontinuing antithrombotics before the surgery and has classified interventions based on the risk of bleeding and hematoma. While they have shown that the incidence of hematoma is rare, discontinuing antithrombotics might increase the risk of thrombotic events as well as stroke, pulmonary emboli, heart attacks, and even death. On the other hand, the hematoma and bleeding reported in the Kaye et al. study are procedural and not specifically related to the blocking method. Most of the reported cases of hematoma or bleeding had occurred during procedures like acupuncture, spinal cord, and interlaminar epidural interventions. In the systematic review of Takaschima et al., the findings of all 5 included studies revealed that there were no notable disparities in the occurrence of mild to moderate hemorrhagic complications among patients who utilized antithrombotic medications (such as aspirin, clopidogrel, and warfarin) and those who did not. Additionally, both groups exhibited extremely low rates of severe hemorrhagic complications which implies that needle blocks are generally considered safe even for individuals taking antithrombotic drugs.In Kietaibl et al.’s research, they found that for certain medical procedures like neuraxial procedures or peripheral nerve blocks, it’s crucial to adhere to specific timeframes when giving antithrombotic drugs. This is particularly important when there’s a higher risk of bleeding. However, for peripheral nerve blocks with a lower risk of bleeding, these timeframes do not need to be followed.

**Figure-1:**
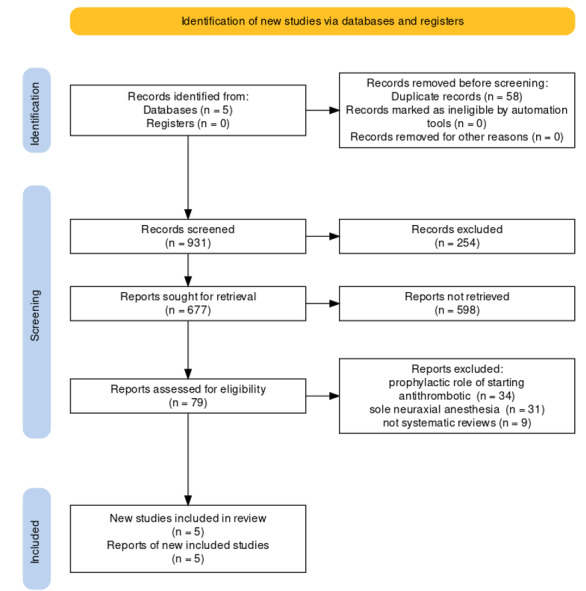
Figure-1comperhansive study Prisma flowchart of selected studies in this study, drawn by online tool [21]

Evidence Synthesis

The schematic in Figure-2 shows the summary of evidence. There is no evidence of puncture site bleeding complications in head and neck punctures except in a rare case showing hematoma incidence after the Infraclavicular brachial plexus block reviewed in the Tsui et al. study. But, a deep cervical plexus block is not recommended.

**Figure-1:**
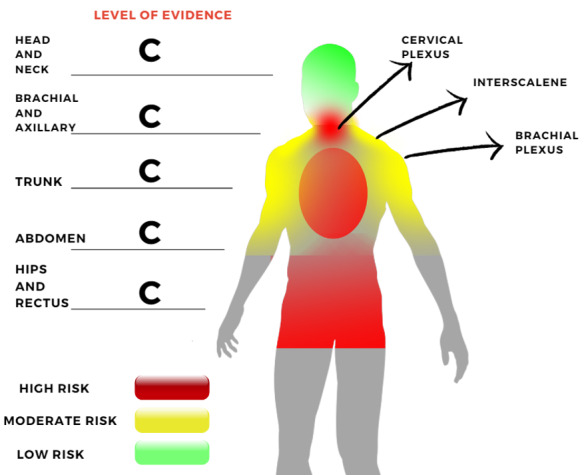
Figure-2The schematic summary of the risk of bleeding in peripheral nerve blocks

**Table:** Table1. Features of the Studies that were Considered and Included in the Review

ID	Year	Study Design	participants	n included studies	Quality*	Main Findings
Tsui et al. [17]	2019	Systematic evidence review and expert consensus	Peripheral nerve blocks and interfacial plane blocks	Not specified	low	Categorized based on risk
Joubert et al. [18]	2019	Systematic review	Peripheral nerve blocks	24	mod	Bleeding complications were rare (incidence: 0.82%)
Kaye et al. [20]	2019	Best evidence synthesis	Interventional pain management procedures	14	low	Risk stratification, discontinuation of medication, and other considerations
Takaschima et al. [19]	2016	Systematic Review	Needle-based ophthalmic regional anesthesia	5	mod	No significant bleeding complications reported
Kietaibl et al. [16]	2022	Systematic literature search, Delphi process	Peripheral nerve and neuraxial blocks	47	high	Strong consensus (>90%) in 57.5% of recommendations, consensus (75 to 90%) in 42.5%; Limited number and quality of clinical studies, GRADE C evidence throughout

^*^Mod, moderate

In Joubert et al. review of six observational studies from 65 bleeding complications of 9688 peripheral nerve blocks non were related to interscalene blocks; while there was a case report of hematoma in interscalene blocks. In Tsui et al. study there were three case reports of hematoma incidence in interscalene blocks and 6 reports of spinal injury. On the other hand, Kietaibl et al. had generally considered interscalene blocks as superficial blocks that might not have a clinically significant complication, which seems not to be logical. In the case of brachial plexus blocks, supraclavicular brachial plexus block was associated with hemothorax as reviewed by Kietaibl et al. while other studies did not mention any hematoma incidence. But, infraclavicular brachial plexus blocks are not favored by both Kietaibl et al. or Tsui et al. There were 6 case reports and 0.2% incidence of minor hematoma in axillary brachial plexus blocks based on Tsui et al. and Joubert et al. review.

Abdomen blocks, as well as the Transversus abdominis plane (TAP) block, are shown to have a higher number of case reports of hematoma in the literature (10 case reports included in Tsui et al. review). Similarly, Ilioinguinal bloks might also cause hematomas [17]; while categorized in superficial blocks in Kietaibl et al. study. Tsui et al. was the sole study that evaluated rectus sheath block with 2 reported cases. There is high-level evidence for Pectoral nerve (PECS) blocks, where there is a relatively high risk of bleeding and hematoma in this case (Tsui et al. and Joubert et al.). Tsui et al. and Kietaibl et al. considered Paravertebral and Intercostal blocks as high risk; while Kietaibl et al. bring the notion that as no hematoma is described in paravertebral procedures where ultrasonography is supplemented, further research might be needed in this case.

## Discussion

According to the recent guidelines from the American Society of Regional Anesthesia, it’s recognized that the clinical outcomes related to bleeding after regional anesthesia procedures can vary. These variations depend on factors like the procedure’s location, the patient’s body size, the compressibility of the site, their underlying health conditions, and their anticoagulation status. However, these guidelines haven’t specified recommendations for peripheral nerve blocks [22]. In the research conducted by Chelly et al., a group of skilled anesthesiologists who specialize in orthopedic anesthesia and peripheral nerve blocks conducted a review regarding the combination of thromboprophylaxis and peripheral nerve blocks in orthopedic surgery. However, it’s important to note that this review wasn’t a systematic examination of the existing literature [23]. The evidence from their study suggests that both thromboprophylaxis and peripheral nerve blocks, particularly continuous techniques, can be beneficial for patients undergoing significant orthopedic surgery. It’s worth mentioning that major bleeding, including retroperitoneal hematoma, can be a complication of both of these treatments. Nevertheless, the review identified only four case reports of major bleeding incidents in patients who received both thromboprophylaxis and peripheral nerve blocks during the period from 1997 to 2012.

This study was an umbrella review of systematic studies that resulted in partially identifying high-risk sites of the body for peripheral nerve blocking; while we aspired to perform meta-analyses with a network meta-analysis (NMA) approach to also evaluate the role of anticoagulant agent type. However different systematic reviews and meta-analysis studies have focused on similar aims in other different clinical settings. De Carlo et al. found that ASA plus PY2P2 inhibitor increases the bleeding risk [24], but their patients were peripheral artery disease subjects. This was also confirmed for single ASA therapy [25]. Pelliccia et al. found that the risk of bleeding in dual antiplatelet therapy is high while is dynamic in time passing the initiation of the treatment regimen. But in our study, we did not address this time measure [26], as well as the Bouget et al. [27].

In atrial fibrillation patients, there was no difference between different groups of oral and direct oral anticoagulants for the chance of procedural bleeding [28]. Wolfe et al. found that different doses of dabigatran as a P2Y12 inhibitor would have different bleeding statuses [29].

## Conclusion

In conclusion, our study findings highlight the importance of careful consideration and individualized risk assessment when selecting and performing specific blocks and procedures. Head and neck punctures generally do not pose a significant risk of bleeding complications, except for rare cases associated with Infraclavicular brachial plexus block. A deep cervical plexus block is not recommended due to potential complications. Interscalene blocks have conflicting findings, with some reports of hematoma incidence and spinal injury. However, further research is needed to clarify the risk profile of paravertebral blocks.

## Acknowledgment

We extend our sincere appreciation to the Clinical Research Development Unit at the Peymanieh Educational and Research Center, in addition to the Therapeutic Center associated with Jahrom University of Medical Sciences, for their invaluable contributions, guidance, and partnership in our endeavors. Their support has been instrumental in our research efforts (IR.JUMS.REC.1401.129).

## Conflict of Interest

There are no conflicts of interest in this study.
